# Direction- and Angle-Assisted Buttonhole Cannulation of Arteriovenous Fistula in Hemodialysis Patients: A Multicenter Randomized Controlled Trial

**DOI:** 10.1016/j.xkme.2021.10.006

**Published:** 2021-12-01

**Authors:** Marit I. Rønning, Willem P. Benschop, Marius A. Øvrehus, Maria Hultstrøm, Stein I. Hallan

**Affiliations:** 1 Department of Nephrology, St. Olav Hospital, Trondheim, Norway; 2 Department of Medicine, Innlandet Hospital, Lillehammer, Norway; 3 Department of Clinical and Molecular Medicine, Faculty of Medicine and Health Sciences, Norwegian University of Science and Technology, Trondheim, Norway

**Keywords:** Angle and direction, arteriovenous fistula, buttonhole, cannulation, guidelines, hemodialysis, pain, randomized clinical trial, skin markings, successful placement

## Abstract

**Rationale & Objective:**

Arteriovenous fistula cannulation with the buttonhole technique is often preferred by patients but has been associated with an increased infection risk. Guidelines disagree on whether it should be abandoned, thus we assessed a technologically simple method to facilitate gentler arteriovenous fistula cannulation with potentially less discomfort and damage to the epithelial lining of the buttonhole tract.

**Study Design:**

8-week, prospective, open-label, randomized controlled trial.

**Setting & Participants:**

Patients with buttonhole tracts receiving hemodialysis at 7 dialysis centers in Norway were randomized to the intervention group (43 patients, 658 cannulations) or control group (40 patients, 611 cannulations).

**Intervention:**

Direction and angle of the established buttonhole tract were marked on the forearm skin in the intervention group, whereas the control group had no structured cannulation information system.

**Outcomes:**

The primary outcome was successful cannulation, defined as correct placement of both blunt needles at the first attempt without needing to change needles, perform extra perforations, or reposition the needle. The secondary outcomes were patient-reported difficulty of cannulation (verbal rating scale: 1 = very easy, 6 = impossible) and intensity of pain (numeric rating scale: 0 = no pain, 10 = unbearable pain).

**Results:**

After a 2-week run-in period, successful cannulation was achieved in 73.9% and 74.8% of the patients in the intervention and control groups, respectively (relative risk [RR], 0.99; 95% CI, 0.87-1.12; *P* = 0.85). However, the probability of a difficult arterial cannulation (verbal rating scale, 3-6) was significantly lower in the intervention group (RR, 0.69; 95% CI, 0.55-0.85; *P* = 0.001). There were no improvements for venous cannulations. Furthermore, the probability of a painful cannulation (numeric rating scale, 3-10) was lower in the intervention group (RR, 0.72; 95% CI, 0.51-1.02; *P* = 0.06).

**Limitations:**

Unable to evaluate hard end points such as infections and thrombosis owing to the small sample size.

**Conclusions:**

Marking direction and angle of cannulation did not improve cannulation success rates; however, patients more often reported an unproblematic procedure and less pain.

**Funding:**

None.

**Trial Registration:**

ClinicalTrials.gov (NCT01536548).

## Graphical abstract

**Figure:**
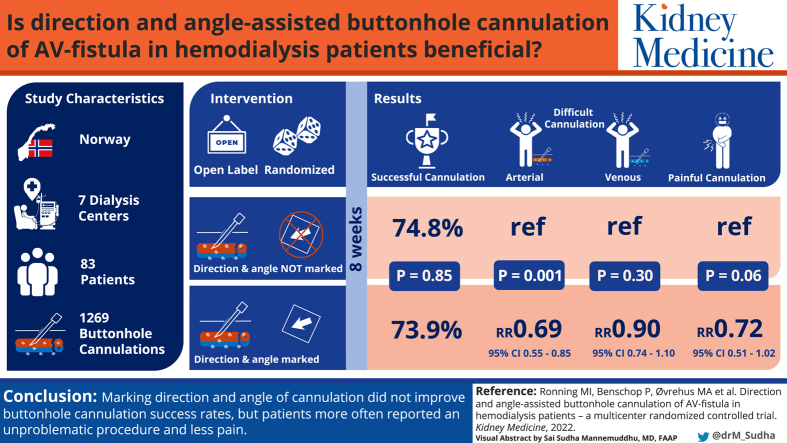

**Plain-Language Summary**

Many patients prefer the use of the buttonhole technique (ie, dialysis needles inserted exactly the same way every time) because it reduces discomfort. However, it can also increase the risk of infection. We tested whether skin markings of needle direction, angle, and rotation for the individual patient could result in less traumatic cannulations. We found that although the nurses did not improve their rate of correct placement on the first attempt, the cannulation was gentler: patients more often reported that the cannulation was “very easy or easy,” and they also reported less pain during cannulation. Less discomfort during dialysis is important for patients, and whether gentler cannulation also reduces infection risk should be tested in a new and larger study.

Chronic kidney disease prevalence is 10%-13% worldwide,^1^, ^2^, ^3^ and the number of patients who start receiving dialysis has increased strongly over the past decades.^4^ However, even after more than 50 years and millions of dialysis treatments, establishing and maintaining a functional blood access is still problematic, and new devices, biological approaches, and novel access techniques are therefore highly needed.^5^

An arteriovenous fistula (AVF) is the preferred access owing to fewer complications, lower mortality, and lower costs than other access types.^6^^,^^7^ There are, however, different techniques for cannulating the AVF. In area cannulation, the same area is always punctured; however, this technique is not recommended because of the high risk of aneurysm formation.^6^^,^^7^ The buttonhole technique involves inserting the needles always in the exact same spot, and several studies have found that it leads to less pain and fewer aneurysms, making it the preferred technique for many patients. However, studies have also found that the buttonhole technique is associated with more frequent *Staphylococcus aureus* infections and no clear advantages.^8^^,^^9^ Recent US guidelines advocate the rope-ladder technique^7^ (ie, systematically changing the needle placement sites for each dialysis), whereas the European 2020 guidelines suggest using either the rope-ladder or the buttonhole technique depending on local expertise and AVF characteristics. Practicing the rope-ladder technique can be difficult in patients with short cannulation segments, leaving buttonhole as the preferred technique in these patients. Furthermore, the European guidelines also highlight the underutilized potential of antiseptic measures and practical aspects of the cannulation procedure to reduce infection risk.^6^ Likewise, weighing a certain daily benefit of less pain against an uncertain future benefit such as avoiding an infection is also a difficult discussion.

The buttonhole technique requires the needle to be inserted with exactly the same angle, direction, and depth every time. It typically takes 6-12 cannulations with a sharp needle to establish the fibrous channel that is sealed with a scab. Later, the nurse uses a blunt needle to avoid damage to the established channel; however, this can be challenging owing to frequent personnel turnover and the fact that cannulation is a skill that requires a large amount of clinical practice before a reasonable competence is developed. A limited number of studies have suggested various methods and devices to improve AVF cannulation success rates. Point-of-care ultrasound-guided cannulation has reduced the number of miscannulations^10^^,^^11^; however, clinical uptake has been limited and the technique is probably less useful for buttonholes. Other suggestions include implantable metal devices to guide the cannulation of deep fistulas, plastic cannulas, and cannulation maps and skin markings of the fistula; however, the efficacy is, in general, not well documented.^12^, ^13^, ^14^

The overall goal for patients and caregivers is healthy and minimally traumatized access with longevity, few complications, and minimal pain and discomfort for the patient. The cannulation technique is one of the major determinants for achieving this goal; however, our knowledge on this topic is limited. The aim of this randomized clinical trial was, therefore, to study whether patient-specific skin markings of buttonhole cannulation direction and angle could facilitate a gentler needle placement as measured by improved success rates and reduced patient discomfort.

## Methods

### Study Design and Randomization

**Figure 1:**
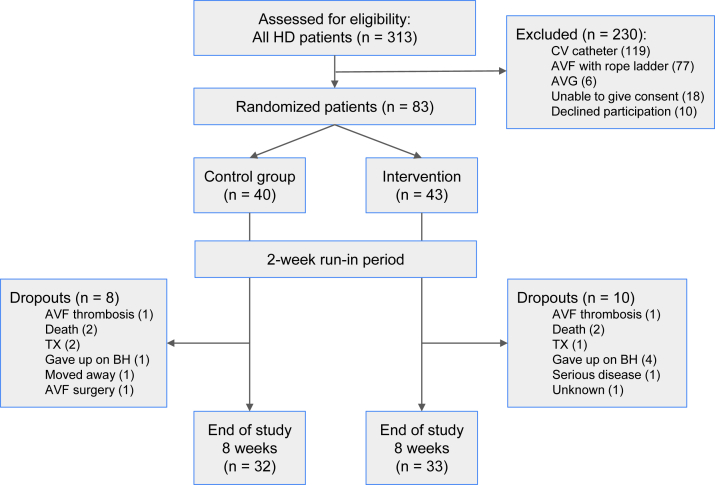
Flowchart describing study design, exclusion criteria, and patient numbers. Abbreviations and definitions: AVF, arteriovenous fistula; AVG, arteriovenous graft; BH, buttonhole; CV, central venous; HD, hemodialysis; TX, kidney transplantation.

All patients with established buttonhole tracts for AVF cannulation receiving hemodialysis at 7 dialysis centers in Norway were eligible for inclusion. All patients, therefore, had a matured AVF, and buttonhole cannulation had been done by registered nurses for at least 2 weeks. These centers constitute the national dialysis access group and represent all geographic regions of Norway, both urban and rural. Exclusion criteria were arteriovenous grafts, age younger than 18 years, inability to communicate well in Norwegian, or inability or unwillingness to sign an informed consent (Fig 1).

There are no national guidelines on AVF cannulation in Norway. Buttonhole cannulation has been the most popular technique in most centers over the last 10-15 years, and the decision is often taken after discussions between the patient and the nurses. There is a strong focus on infection control with cannulation following the principles of sterile technique (eg, sterile gloves, face masks, sterile surgical drapes, thorough disinfection of patient arm, careful removal of buttonhole tract scab). All study centers report to the Norwegian renal quality registry and perform well on the 22 quality indicators^15^; however, infection rates by AVF cannulation technique are not routinely reported.

The study coordinator at each center used the WebCRF2 Version 1.2 (Unit for Applied Clinical Research, Norwegian University of Science and Technology, Norway) to include the patient and register patient and AVF baseline characteristics, and the nurses used optically readable paper schemes to register outcome information at each dialysis session during the study period (see the next subsection). Patients were individually computer-randomized 1:1 to the intervention or control group using block randomization with a variable block size of 6, 8, or 10 patients. The first block consisted of 10 patients, with 5 patients randomized to the intervention and 5 to standard care. The subsequent block sizes randomly varied among 6 (ie, 3 + 3), 8 (ie, 4 + 4), or 10 (ie, 5 + 5) patients, with one block created when the former was completed. There was no stratification based on the study center or other baseline characteristics.

After randomization, the first 2 weeks of the 8-week study period were defined as a run-in or training period in which the patients were cannulated using either the intervention or the standard cannulation technique to allow nurses and patients to become accustomed to the new cannulation aid of the intervention group. This was done to reduce the impact of learning the new technique, as relevant learning curves typically seem to flatten after 20 procedures,^16^^,^^17^ which equals 2 weeks of work for most nurses. All cannulations were done by registered nurses.

We estimated a 10 percentage points increase of our primary outcome (eg, successful cannulation rates of 0.80-0.90) as the minimal meaningful effect size. Although no previous research was available on the intraclass correlations (ρ), clinical experience indicates a rather weak correlation (ρ < 0.20). Based on an α value of 0.05, power of 0.80, and cluster size of 18 hemodialysis sessions (3 sessions per week) per patient with ρ = 0.10, sample size calculations indicated that a sample size of 30 patients (21-49 for ρ of 0.05-0.20) was needed for each study group (Stata command “power twoproportions”). These estimates should, however, be considered vague approximations.

The trial adhered to the Declaration of Helsinki, was registered at ClinicalTrials.gov (NCT01536548), and was approved by the Regional Committee for Medical and Health Research Ethics for Central Norway before starting the study (REK 2011/2544). All patients gave written informed consent for inclusion.

### Data Collection and Outcome Definitions

Patient baseline data included age, sex, height, and weight as well as information on comorbid conditions such as diabetes, stroke, angina pectoris, myocardial infarction, and anticoagulation medication. Characteristics of AVF were also registered (placement, depth, diameter, length, tortuosity, flow, age, and time since buttonhole establishment). The primary outcome was nurse-reported “successful cannulation” of each dialysis, defined as correct placement of 2 blunt needles at the first attempt (ie, without needing to change needles, perform extra perforations, or reposition the needle). Secondary outcome measures were patient-reported difficulty and pain at the first cannulation attempt. Patient-reported cannulation difficulty was reported on a 1-6 verbal rating scale for every arterial and venous cannulation (1 = very easy, 2 = easy, 3 = satisfactory, 4 = a bit difficult, 5 = difficult, and 6 = impossible). Anxiety and pain were evaluated once a week on a verbal rating scale ranging from 0 (no fear or pain) to 10 (unbearable fear or pain).

### Intervention

**Figure 2:**
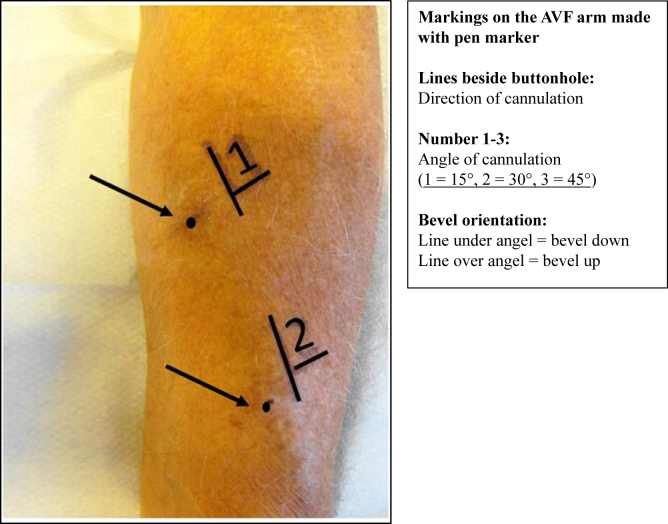
Skin markings (arrows) made on the arm in the intervention group to help cannulate the arteriovenous fistula with the same direction and angle used for developing the buttonhole tract. Abbreviation: AVF, arteriovenous fistula.

The intervention group was cannulated with the aid of a skin drawing that specified the direction and angle of cannulation and the needle bevel orientation for the individual patient, whereas the control group was cannulated using the standard practice without formal aids or structuration to guide cannulation. An experienced registered nurse familiar with the patient’s AVF did the markings with a skin marker pen on the patients in the intervention group. For both the arterial and venous cannulation holes, the direction of cannulation was marked with a line (Fig 2). The angle of cannulation was marked with a number (1 = 15°, 2 = 30°, and 3 = 45°) consistent with local practice and because most experts and guidelines recommend a cannulation angle of 25°-30° for AVFs.^18^^,^^19^ The needle bevel orientation was marked with a short line below or above the needle angle number to indicate bevel up or down, respectively. We used skin marker pens similar to those that have been used for many years in radiotherapy, and each patient in the intervention group got their own pen owing to infection prevention considerations.^20^ The patients were encouraged to be gentle when washing the arm to avoid markings disappearing between the hemodialysis treatments. The markings were to be refreshed once a week or whenever needed. There was no extra training of the nurses, and they rotated randomly between the intervention and control patients as usual; therefore, except for the skin markings, patients in the intervention and control groups were treated identically. Some centers might have information on the direction of the buttonhole tract in the patient files; however, this information was not organized in a systematic way and not automatically presented to the nurse cannulating the patients in the control group.

### Statistical Analyses

We used mean (1 standard deviation), median (25th-75th percentiles), and percentages to describe baseline characteristics. Statistical analyses were performed according to the intention-to-treat principle. We used generalized estimation equations (GEEs) to study whether our intervention influenced various outcomes. The GEE is a type of regression analysis suitable for longitudinal studies in which each patient is evaluated several times such that the outcomes are not independent.^21^ The method can evaluate both dichotomous and continuous outcomes and is, in general, based on rather few and soft assumptions. We used a model selection method (“quasi-likelihood under the independence model criterion,” Stata command “qic”) to select the best working correlation structure for the GEE regression.^22^ Furthermore, simulation studies have shown that for small-to-moderate groups (n < 50), effect and variability estimates are only weakly influenced even if the correlation structure is misspecified.^23^ We used the independent correlation structure for all outcomes, and the Poisson and gamma distributions were used for dichotomous and continuous outcomes, respectively. Based on clinical experience and the literature, we prespecified age, stroke, the nurse’s experience with the patient’s AVF, buttonhole age, and AVF diameter and depth as covariates to adjust for general peripheral vessel disease risk, local AVF factors, and factors related to the cannulation per se. The association with perceived pain was adjusted for patient anxiety and the use of local anesthetics. The significance level was set at 0.05, and we used STATA 14 (StataCorp LP) for all analyses.

## Results

We screened 313 patients receiving hemodialysis from 7 Norwegian dialysis centers, and 83 patients were eligible for inclusion. Of these, 40 patients were randomized to the control group and 43 to the intervention group (Fig 1); however, 2 patients from each group dropped out during the 2-week run-in period. The number of patients per center ranged from 4 to 22, and a total of 120 nurses worked at these centers. No patients were lost to follow-up. The mean age was 66 years, 70% of the patients were men, and diabetes mellitus and cardiovascular disease were highly prevalent at 32% and 27%, respectively. Median time since AVF and buttonhole creation was 9 and 5 months, respectively. Further details on patient and AVF characteristics by study group are given in Table 1.

**Table 1:** Baseline Characteristics of Patients and Their Arteriovenous Fistulas by Randomization Group

	Control Group (n = 38)	Intervention Group (n = 41)
Age, y	63.1 ± 15.7	68.2 ± 11.7
Male sex (%)	24 (63.2)	31 (75.6)
Body mass index, kg/m^2^	25.4 ± 4.8	27.5 ± 6.0
Diabetes mellitus (%)	14 (36.8)	11 (26.8)
Stroke (%)	3 (7.9)	5 (12.2)
Myocardial infarction (%)	5 (13.2)	5 (12.2)
Angina pectoris (%)	4 (10.5)	6 (14.6)
Hypertension (%)	19 (50.0)	22 (53.7)
Hemodialysis, sessions/wk	3.1 ± 0.6	3.1 ± 0.5
AVF		
Deep (%)	9 (23.7)	7 (17.1)
Diameter < 6 mm (%)	7 (18.4)	13 (31.7)
Length < 8 cm (%)	10 (26.3)	12 (29.3)
Flow < 600 mL/min (%)	7 (18.4)	11 (26.8)
Time since AVF creation, mo	7 (2-22)	11 (4-22)
Time since buttonhole creation, mo	3 (1-11)	5 (2-12)

*Note*: Dichotomous data are expressed as numbers (%). Continuous data are expressed as mean ± 1 SD; time since AVF and buttonhole creation is expressed as median (25th-75th percentiles).

Abbreviation: AVF, arteriovenous fistula.

**Figure 3:**
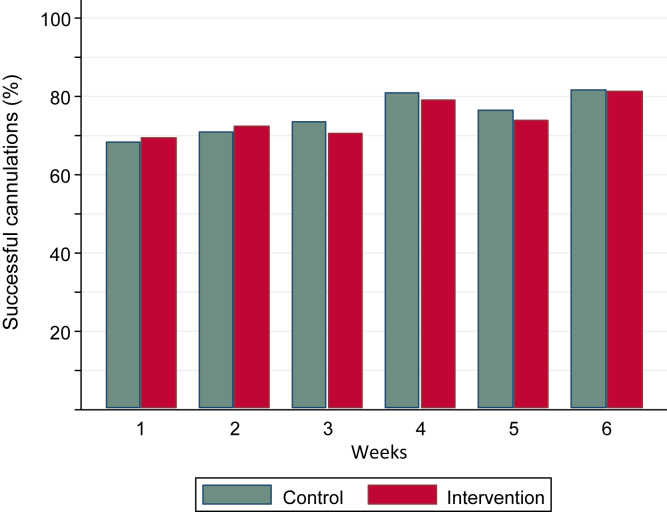
Observed rate of successful cannulation defined as correct placement of both arterial and venous blunt needles at the first attempt in the control and intervention groups over the study period (week 1-6, n = 1,269).

We evaluated 1,269 cannulations over the 6 weeks following the 2-week run-in period. The success rate for correct placement of 2 blunt needles at the first attempt was 74.8% and 73.9% in the control and intervention groups, respectively. Most of the unsuccessful cannulations were completed with the help of another nurse to reposition the needle or with the second attempt using the same buttonhole tract. A new cannulation site and the use of a sharp needle were necessary to start dialysis in only 5% of the cases. Figure 3 shows the cannulation success rate by study week. There were only small (<5%) and inconsistent differences in the cannulation success rates between the groups over the study period. Likewise, there was no statistically significant overall difference between the groups based on GEE regression analyses accounting for repeated testing of the patients over the 6-week study period (relative risk [RR] for successful cannulation, 0.99; *P* = 0.85). Adjustment for clinically relevant covariates (age, stroke, the nurse’s experience for a particular AVF, time since buttonhole creation, and AVF diameter and depth) did not change the effect (Table 2).

**Table 2:** Effect of Study Intervention Versus the Control Group for Various Outcomes Evaluated With Regression Analyses

Dichotomous outcomes	Crude (n = 1,269)			Adjusted (n = 1,233)		
	RR	95% CI	*P* value	RR	95% CI	*P* value
Successful cannulation (no/yes)	0.99	0.87 to 1.12	0.85	0.98^a^	0.86 to 1.12	0.78
Difficult arterial cannulation (1-2/3-6)	0.69	0.55 to 0.85	0.001	0.76^a^	0.60 to 0.96	0.02
Difficult venous cannulation (1-2/3-6)	0.90	0.74 to 1.10	0.3	0.96^a^	0.77 to 1.19	0.69
Painful cannulation (0-2/3-10)	0.72	0.51 to 1.02	0.06	0.66^b^	0.45 to 0.98	0.04

Continuous outcomes	Beta	95% CI	*P* value	Beta	95% CI	*P* value
Arterial cannulation difficulty (1-6)	−0.27	−0.41 to −0.14	<0.001	−0.21^a^	−0.35 to −0.08	0.002
Venous cannulation difficulty (1-6)	−0.01	−0.17 to 0.15	0.91	0.00^a^	−0.16 to 0.16	0.98
Pain intensity at cannulation (0-10)	−0.28	−0.61 to −0.05	0.09	−0.52^b^	−0.83 to −0.21	0.001

*Note*: We used generalized estimation equations with Poisson distribution for dichotomous outcomes and gamma distribution for continuous outcomes, canonical link functions (log and reciprocal transformation, respectively), and an independent correlation structure.

Abbreviations: CI, confidence interval; RR, relative risk.

a Adjusted for age, stroke, nurse’s experience with the patient’s arteriovenous fistula, buttonhole age, and arteriovenous fistula diameter and depth.

b Adjusted for anxiety and local anesthetics. Covariates were selected by clinical relevance.

**Figure 4:**
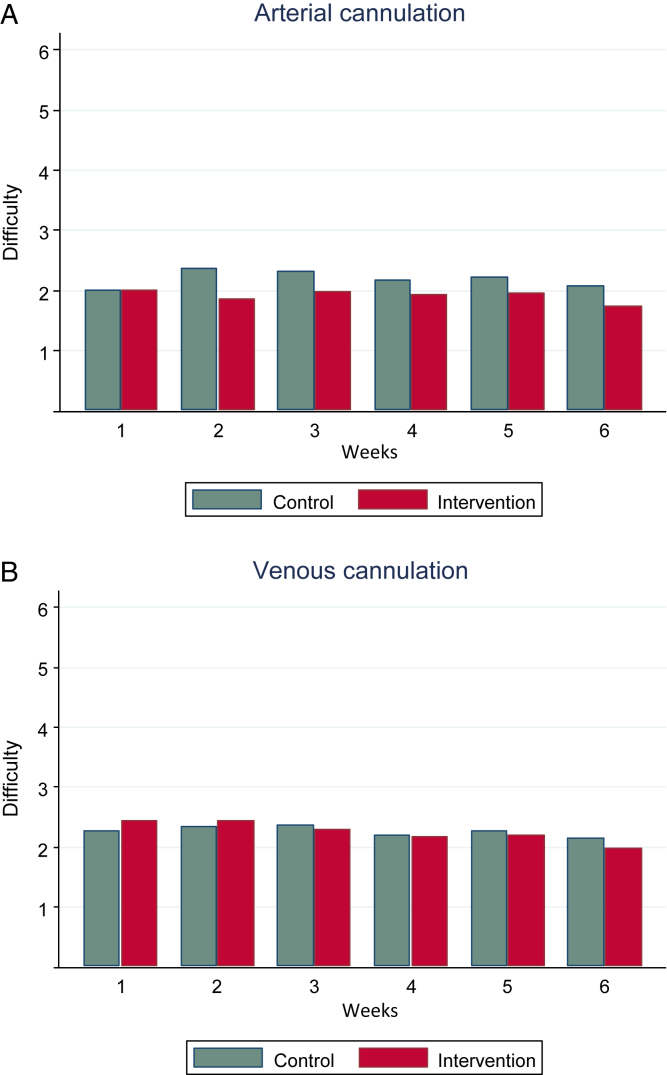
Patient-reported cannulation difficulty (1 = very easy, 6 = impossible) for (A) arterial and (B) venous buttonhole in controls versus intervention groups over the study period (week 1-6, n = 1,269).

We subsequently tested the effect of skin marking by assessing patients’ evaluation of the difficulty of cannulation (1 = very easy, 6 = impossible). Figure 4A and B displays the mean difficulty level for the arterial and venous cannulations, respectively, over the study period. The intervention group had a lower mean arterial cannulation difficulty throughout the study period (Fig 4A). The mean difficulty was 2.18 in the control group compared with 1.91 in the intervention group (difference, −0.27). A similar effect was found with regression analysis after accounting for repeated testing (β coefficient, −0.27), and this intervention effect was highly significant (*P* < 0.001). Defining the upper tertile of responses as a difficult cannulation (ie, difficulty, 3-6), Table 2 shows that RR of a difficult cannulation decreased with 31% in the intervention group compared with the control group (RR, 0.69; *P* = 0.001). Adjustment for the same clinically relevant covariates as mentioned above did not change the results for either the continuous or the dichotomous outcome. Venous cannulations did not improve in the intervention group (RR, 0.90; *P* = 0.30).

**Figure 5:**
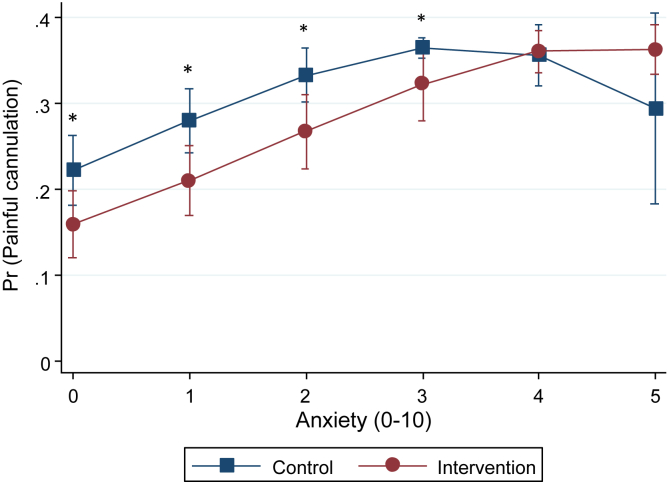
Probability of painful cannulation by level of anxiety in the control group versus the intervention group after adjusting for the use of local anesthesia and time since arteriovenous fistula creation. Error bars represent 95% confidence intervals (n = 1,269). Abbreviation: Pr, probability. ∗, *P* < 0.05.

Pain was assessed at 1 dialysis session per week throughout the study period with a combined response for the arterial and venous cannulation (0 = no pain, 10 = unbearable pain). Figure 5 displays the probability of a painful cannulation, defined as the upper tertile of responses (ie, pain, 3-10), in the intervention versus control group by levels of anxiety with adjustments for local anesthetics. There was a rather constant effect of the intervention over anxiety levels 0-4. Very few patients had higher anxiety levels (>4), and estimates in this range were unstable and not significantly different. Regression analysis in Table 2 showed that the intervention group had crude and adjusted RRs for painful cannulation of 0.72 and 0.66, respectively (*P* = 0.06 and *P* = 0.04). On the continuous scale, the intervention decreased the mean pain with 0.28 and 0.52 points, respectively (*P* = 0.09 and *P* = 0.001).

Two patients, one from each randomization group, showed signs of minor infection around the buttonhole tract. No systemic infections were reported during the study. Other adverse events reported from the control and intervention groups, respectively, were AVF dysfunction needing percutaneous transluminal angioplasty treatment (2 + 4 patients), AVF occlusion due to thrombosis (1 + 1 patients), and death (2 + 2 patients). Patients also left the study due to kidney transplantation (2 + 1 patients), reduced general health (0 + 1 patient), moving to a nonparticipating dialysis center (1 + 0 patient), or other reasons (2 + 5 patients).

## Discussion

We tested whether a skin marking to help maintain the established cannulation direction and angle could improve buttonhole cannulation of AVFs. The intervention did not improve the nurses’ ability to cannulate both the arterial and venous sites with blunt needles at the first attempt. However, the patients in the intervention group significantly more often reported that the arterial cannulations were “very easy” or “easy.” They also reported a significantly lower level of pain during the cannulations than that reported by the control group.

US guidelines advise against buttonhole (constant site) cannulation owing to reports of increased risk of *S aureus* infections.^7^ However, the technique is still preferred by many patients,^24^, ^25^, ^26^ and the European and British guidelines rather focus on the advice to reduce the infection problem because the data on infectious risk have varied greatly.^6^^,^^27^ We, therefore, studied how to further improve the technique as this could reduce patient discomfort and potentially also reduce the infection risk.

Patients receiving dialysis have severely decreased quality of life,^28^ and pain is reported as one of the essential outcomes for the majority of patients with chronic kidney disease.^29^^,^^30^ Several studies have found that patients cannulated with the buttonhole technique experience less pain compared with the rope-ladder technique.^31^ However, not all studies have confirmed this.^32^ Our simple intervention could improve the results further, and this would be an important and immediate benefit. Many patients are risk-averse and prefer outcomes with low uncertainty to outcomes with high utility but high uncertainty; that is, it is better to have the benefit of less pain at most dialysis sessions than to potentially avoid an infection next year. Physicians tend to give higher weightage to hard outcomes such as infections, and this could bias evaluations of the buttonhole technique.^33^ It is unclear why we did not observe any improvement for the venous cannulation site; however, the venous site is generally held to be more difficult to cannulate owing to a smaller diameter and deeper placement. Patients, in general, felt positive about the skin markings; however, some felt that the markings were an esthetic problem. For long-term use, other methods of information exchange should be explored.

A few studies have investigated the mechanisms behind the increased infection risk associated with the buttonhole cannulation technique.^34^^,^^35^ *S aureus* is highlighted as the major pathogen, with skin and nostrils of both patients and nurses as potential reservoirs. The bacteria are also regularly found in the buttonhole scab and the upper parts of the tract. Our study does not have the power or design to study hard long-term end points such as AVF patency or clinical infections; however, reduced cannulation difficulty and less pain in the intervention group indicate a gentler and more problem-free procedure. Improved cannulation technique will prevent disruption of the epithelial lining of the buttonhole tract as well as the creation of pockets and false tunnels. This may reduce the risk of bacteria penetrating the surrounding tissues and starting an infection.^32^^,^^36^, ^37^, ^38^ Less inflammation, edema, and hematoma may also explain the reduced pain.

There have also been some reports on other methods for improving AVF cannulation. The Venous Window Needle Guide device (Vital Access Corp) was developed for the salvage of noncannulatable AVFs that are otherwise functional. One study reported successful AVF access in 49 of the 51 patients evaluated, and only 1 device was removed because of infection over a 6-month period.^12^ However, it is still a highly invasive procedure with substantial risks that will be used only in carefully selected cases. Plastic cannulas are claimed to reduce AVF damage caused by direct injury or the decubitus of the needle on the wall, which can increase the risk of thrombosis and stenosis of the fistula.^13^ Ultrasound-guided cannulation is probably the most documented and promising method for improved cannulation. A recent systematic review found 21 relevant published studies; however, only 5 studies were primary research publications, and the number of included patients was rather small.^10^ They concluded that there was a lack of robust studies; however, ultrasound-guided cannulation could probably increase the number of successful cannulations and reduce the risk of complications and misalignment of the needles. Regarding the buttonhole method, it has been suggested that such simple handheld ultrasound devices may enhance the ability to develop the needle tracks, which is a difficult and critical part of the buttonhole method.^11^

The current study has several strengths worth mentioning. We used adequate methods with a randomized design and appropriate statistics, the inclusion rate was very high, and there were few dropouts during the study period. This reduced the risk of selection bias and other causes of random errors. Furthermore, a multicenter design improves the generalizability of our study; however, the effect could still be different in other regions outside Northern Europe. All centers had an experienced staff of nurses, and there was a high focus on hygiene and infection control. This makes the study relevant for the current European recommendations of the buttonhole technique for in-center hemodialysis. The study period was long enough to give sufficient time to address real-life clinical practice with rotating nurses and patient adaption to the new intervention. Outcomes were valid and easy to measure with a focus on patients’ evaluations. However, there are also some weaknesses that need to be discussed. We included only a moderate number of participants, thus smaller effect sizes could have been missed (low power, type II error). Likewise, the incidence of hard end points such as AVF survival, local and systemic infections, and patient survival was very low. Our study cannot provide meaningful information on whether the intervention also has effects on these outcomes, and a much larger study with several years of follow-up is needed.

In conclusion, skin markings of direction and angle did not improve the overall buttonhole cannulation success rate. However, patients reported fewer cannulation difficulties and less pain with the new technique. Less traumatic cannulation with less damage to the epithelial lining of the buttonhole tract could potentially reduce infection risk; however, this hypothesis needs to be tested in a larger trial with a longer follow-up period. Currently, the clinical importance of the intervention is to reduce the patients’ daily procedure-related discomfort.
